# Hydrolysis of Fucoidan by Fucoidanase Isolated from the Marine Bacterium, *Formosa algae*

**DOI:** 10.3390/md11072413

**Published:** 2013-07-11

**Authors:** Artem S. Silchenko, Mikhail I. Kusaykin, Valeriya V. Kurilenko, Alexander M. Zakharenko, Vladimir V. Isakov, Tatyana S. Zaporozhets, Anna K. Gazha, Tatyana N. Zvyagintseva

**Affiliations:** 1 G.B. Elyakov Pacific Institute of Bioorganic Chemistry, Far Eastern Branch of the Russian Academy of Sciences, Prospect 100 Let Vladivostok 159, Vladivostok, 690022, Russia; E-Mails: artem.silchenko@yandex.ru (A.S.S.); valerie@piboc.dvo.ru (V.V.K.); rarf@yandex.ru (A.M.Z.); isakov@piboc.dvo.ru (V.V.I.); zvyag@piboc.dvo.ru (T.N.Z.); 2 Far-Eastern Federal University, ul. Sukhanova 8, Vladivostok, 690950, Russia; 3 Research Institute of Epidemiology and Microbiology, Siberian Branch of Russian Academy of Medical Sciences, Selskaya str. 1, Vladivostok, 690087, Russia; E-Mails: niiem_vl@mail.ru (T.S.Z.); angazha@mail.ru (A.K.G.)

**Keywords:** fucoidanase, fucanase, marine bacteria, *Formosa algae*, fucoidan, *Fucus evanescens*

## Abstract

Intracellular fucoidanase was isolated from the marine bacterium, *Formosa algae* strain KMM 3553. The first appearance of fucoidan enzymatic hydrolysis products in a cell-free extract was detected after 4 h of bacterial growth, and maximal fucoidanase activity was observed after 12 h of growth. The fucoidanase displayed maximal activity in a wide range of pH values, from 6.5 to 9.1. The presence of Mg^2+^, Ca^2+ ^and Ba^2+^ cations strongly activated the enzyme; however, Cu^2+^ and Zn^2+^ cations had inhibitory effects on the enzymatic activity. The enzymatic activity of fucoidanase was considerably reduced after prolonged (about 60 min) incubation of the enzyme solution at 45 °C. The fucoidanase catalyzed the hydrolysis of fucoidans from *Fucus evanescens* and *Fucus vesiculosus*, but not from *Saccharina cichorioides*. The fucoidanase also did not hydrolyze carrageenan. Desulfated fucoidan from *F. evanescens* was hydrolysed very weakly in contrast to deacetylated fucoidan, which was hydrolysed more actively compared to the native fucoidan from *F. evanescens*. Analysis of the structure of the enzymatic products showed that the marine bacteria, *F. algae*, synthesized an α-l-fucanase with an endo-type action that is specific for 1→4-bonds in a polysaccharide molecule built up of alternating three- and four-linked α-l-fucopyranose residues sulfated mainly at position 2.

## 1. Introduction

Fucoidans belong to a family of sulfated homo- and hetero-polysaccharides, including polysaccharides composed primarily of sulfated fucose. Galactose, mannose, xylose, rhamnose and glucuronic acid were also found in various fucoidans [1,2]. Brown algae can synthesize not only linear, but also highly branched polysaccharides with species-specific sugar compositions [1,2,3,4], and for this reason, fucoidan structures are extremely diverse. In addition, each species can form different types of fucoidans [5]. The most studied fucoidans in terms of structure are α-l-fucans with either α-1→3-backbones or repeating disaccharide units of α-1→3- and α-1→4-linked fucose residues. Depending on the structure of the main chain, fucoidans as a rule are sulfated at the C4, C2 or both the C2 and C4 positions of the fucose units in the rare cases that sulfate groups can occupy C3 position [6,7,8,9,10]. Fucoidans exist that contain fucose and galactose residues in equal quantity [2], and some fucoidans may be both sulfated and acetylated [7,11].

Fucoidans have diverse biological activities, including anti-tumor [3,12,13], immunomodulatory [14,15,16], antibacterial [17,18], antiviral [19,20,21,22], anti-inflammatory [23], anticoagulant and antithrombotic [24] effects.

The data reported in the literature show that fucoidans possess a wide spectrum of pharmacological activities; however, they cannot be successfully used in the construction of new drugs, because of their high molecular weight and significant problems with polysaccharide standardization. One approach to solving this problem is the use of enzymes to depolymerize the fucoidans.

Only a few studies regarding the isolation and characterization of fucoidanases have been performed. Until now, enzymes that degrade fucoidans were found in the following marine organisms: bacteria [25,26,27], invertebrates [28,29,30,31,32] and some fungi [33]. However, the fucoidanases produced by these organisms have low activities. Information regarding the specificity of fucoidanases, such as the type of glycosidic bond cleaved and the influence of the degree of sulfation of substrate on the catalytic activity, is scarce. The nucleotide sequences of the genes encoding fucoidanases and their deduced amino acid sequences have been published for the *Mariniflexile fucanivorans* SW5 [34] and *Fucanobacter lyticus* SN-1009 fucoidanases only [35], and technologically valuable sources of these enzymes have not yet been found.

In this study, we report the purification and catalytic properties of a new fucoidanase from the marine bacterium, *Formosa algae*.

## 2. Results and Discussion

The bacterial strain, KMM 3553, from the PIBOC FEB RAS collection of marine microorganisms was chosen as the fucoidanase producer based on the screening results [36] and was identified as *Formosa algae* [37]*.* The fucoidanases found in micro- and macro-organisms have an extremely low level of enzymatic activity [1]. Moreover, the activity of these fucoidanases is often lost during the purification process. For this reason, the majority of the studies regarding the catalytic properties of fucoidanases were performed with either extracts or partially purified enzyme preparations. In addition, fucoidans of unknown structure are often used as the substrate. Fucoidanase from *F. algae* (FFA) is an intracellular enzyme with relatively low activity. To detect the products of fucoidan enzymatic hydrolysis, we have used C-PAGE (carbohydrate polyacrylamide gel electrophoresis). It should be noted that there are difficulties in determining the activity of fucoidanase, because there are few hydrolyses. Traditional methods for the detection of polysaccharide hydrolase activity, such as determination of the quantity of reducing sugars in the reaction mixture, are difficult to employ, because of low sensitivity. 

**Figure 1:**
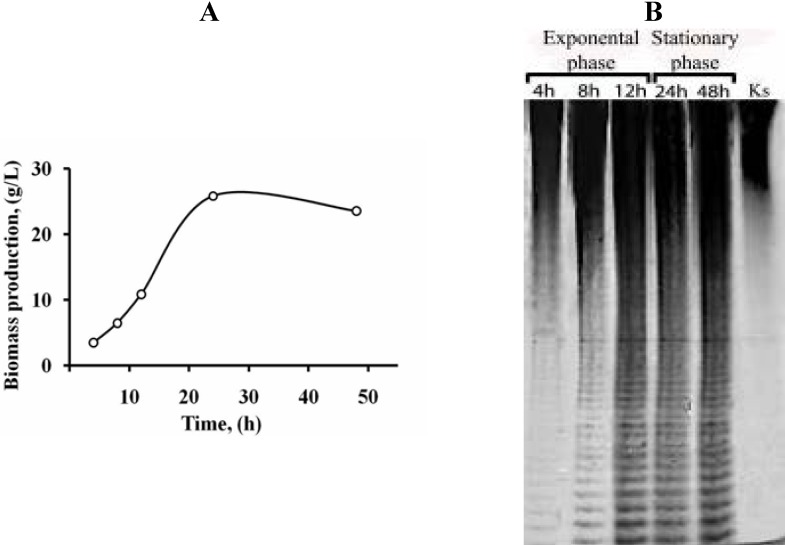
Production of fucoidanase in the bacterial strain, KMM 3553. (**A**) Biomass production; (**B**) Carbohydrate polyacrylamide gel electrophoresis (C-PAGE) electropherogram of hydrolysis products of fucoidan from *F. evanescens* by a cell-free extract of strain KMM 3553 at different growth times. Ks—unhydrolyzed fucoidan.

Fucoidan from *F. evanescens* that was sulfated primarily at C2 was used as the substrate [7]. To detect the sulfated oligosaccharides that were produced during the enzymatic hydrolysis of fucoidan, C-PAGE was used (Figure 1).

The kinetics of bacterial growth in liquid medium and the biosynthesis of fucoidanase were evaluated. The maximum quantity of biomass was observed after 24 h of growth. The enzymatic activity was detected in the microbial mass only and was completely absent in the culture medium. The first appearance of the enzymatic hydrolysis products of fucoidan in a cell-free extract was detected by polyacrylamide gel electrophoresis after 4 h of bacterial growth. The maximal fucoidanase activity was observed after 12 h of growth (Figure 1).

The composition and activity of other *O*-glycoside hydrolases in the cell extracts of *F. algae* were also analyzed. As shown in Table 1 a highly active β-d-glucosidase, *N*-acetyl-β-d-glucosaminidase, and the less active enzymes, alginate lyase and laminarinase (Table 1), were detected. A combination of methods, including anion-exchange chromatography, gel-filtration and ultrafiltration, was selected for the effective isolation of fucoidanase away from the other enzymes.

**Table 1:** Composition and activity of *O*-glycoside hydrolases from bacterial strain, KMM 3553, cell-free extract.

Enzyme substrate	Specific activity (U/mg of protein) *
Alginic acid	6.4
Amylopectin	0
СM-cellulose	0
Laminarin	10.6
Pustulan	0
*p*-Np-*N*-acetyl-β-d-glucosaminide	20.7
*p*-Np-β-d-galactopyranoside	0
*p*-Np-β-d-glucopyranoside	9.5
*p*-Np-β-d-mannopyranoside	0
*p*-Np-α-d-fucopyranoside	0
*p*-Np-sulfate	0

* Specific activity is the amount of enzyme required to release one μmol of glucose or *p*-nitrophenol from the appropriate substrate under standard conditions during 1 min per amount of total protein (mg) in the sample.

**Figure 2:**
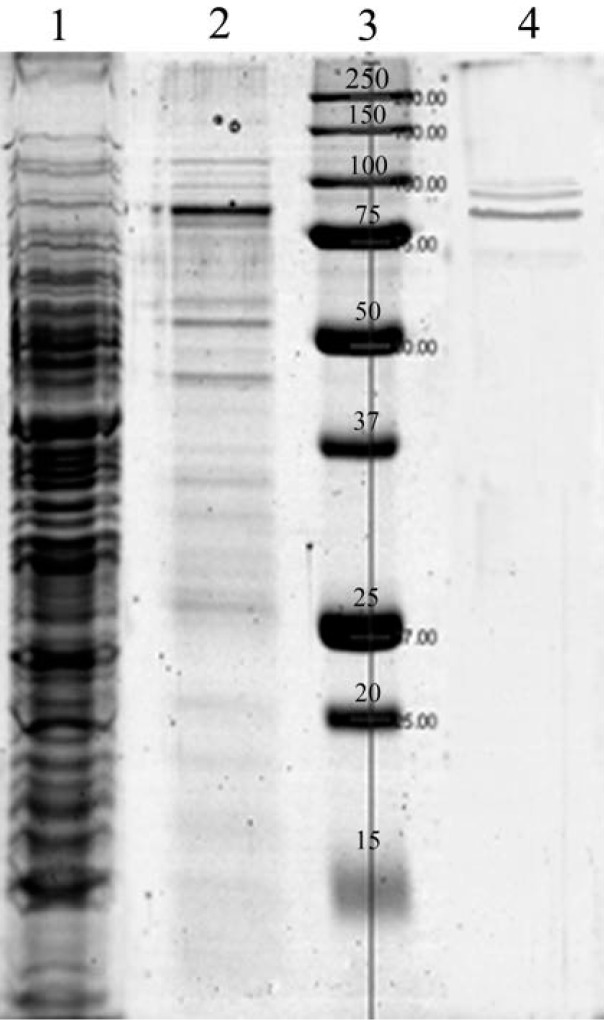
Electropherogram of the fucoidanase isolation. 1—extract, 2—after ion-exchange chromatography, 3—standard and 4—after gel-filtration and ultrafiltration.

The study of fucoidanase properties was performed with the use of a highly purified fucoidanase from the marine bacterium, *F. algae* (FFA). Figure 2 shows an electropherogram of the fucoidanase at the different stages of purification. Only three bands of the proteins on the finishing stage of purification were seen. Furthermore, the fucoidanase was free from enzymes, which is shown in Table 1.

**Figure 3:**
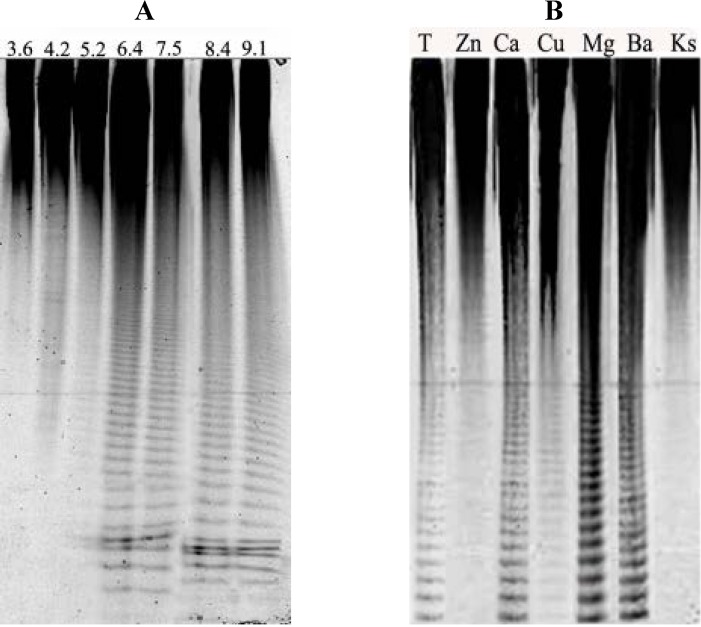
(**A**) Electropherogram of the hydrolysis products of fucoidan produced by fucoidanase from *F. algae* (FFA) at different pH values. The numbers above the line refer to the buffer with a different pH. (**B**) The influence of the different bivalent cations on fucoidanase was monitored by C-PAGE. The cations of bivalent metals are indicated over the lines. T—the control for enzymatic activity without the addition of the bivalent cations. Ks—unhydrolyzed fucoidan.

In most cases, the fucoidanase isolated from marine invertebrates was active in the acidic pH range [32] with the exception of enzymes found in the hepatopancreas of the marine mollusk, *Littorina kurila*. In this organism, two forms of fucoidanase were detected that had an acidic pH-optimum (approximately 5.5) and alkaline (approximately 8) [31]. The fucoidanases isolated from marine bacteria are mostly active in the neutral or slightly alkaline pH range [25,27,38]. FFA showed maximal activity over a wide range of pH values, from 6.5 to 9.1 (Figure 3A), which are more typical for the fucoidanases of marine bacteria.

FFA activity was significantly increased in the presence of the Ca^2+^, Ba^2+^ and Mg^2+^ cations, but the Cu^2+^ cations partially inactivated the enzyme. The complete inactivation of fucoidanase was observed in the presence of Zn^2+^ (Figure 3B). The effect of bivalent metal cations on the enzymatic activity of fucoidanase from the marine bacteria, *F. lyticus* SN-1009 and *Vibrio*. sp. N-5, and fucoidanase from the clam, *Haliotis* sp. [32,38,39], was previously investigated. These enzymes were not metal-dependent; however, the presence of some metal cations affected the fucoidanase activity. The Co^2+^ cations weakly activated the fucoidanase from *F. lyticus* SN-1009, but the Zn^2+^, Cu^2+^ and Hg^2+^ cations had an inhibitory effect on enzyme activity.

**Figure 4:**
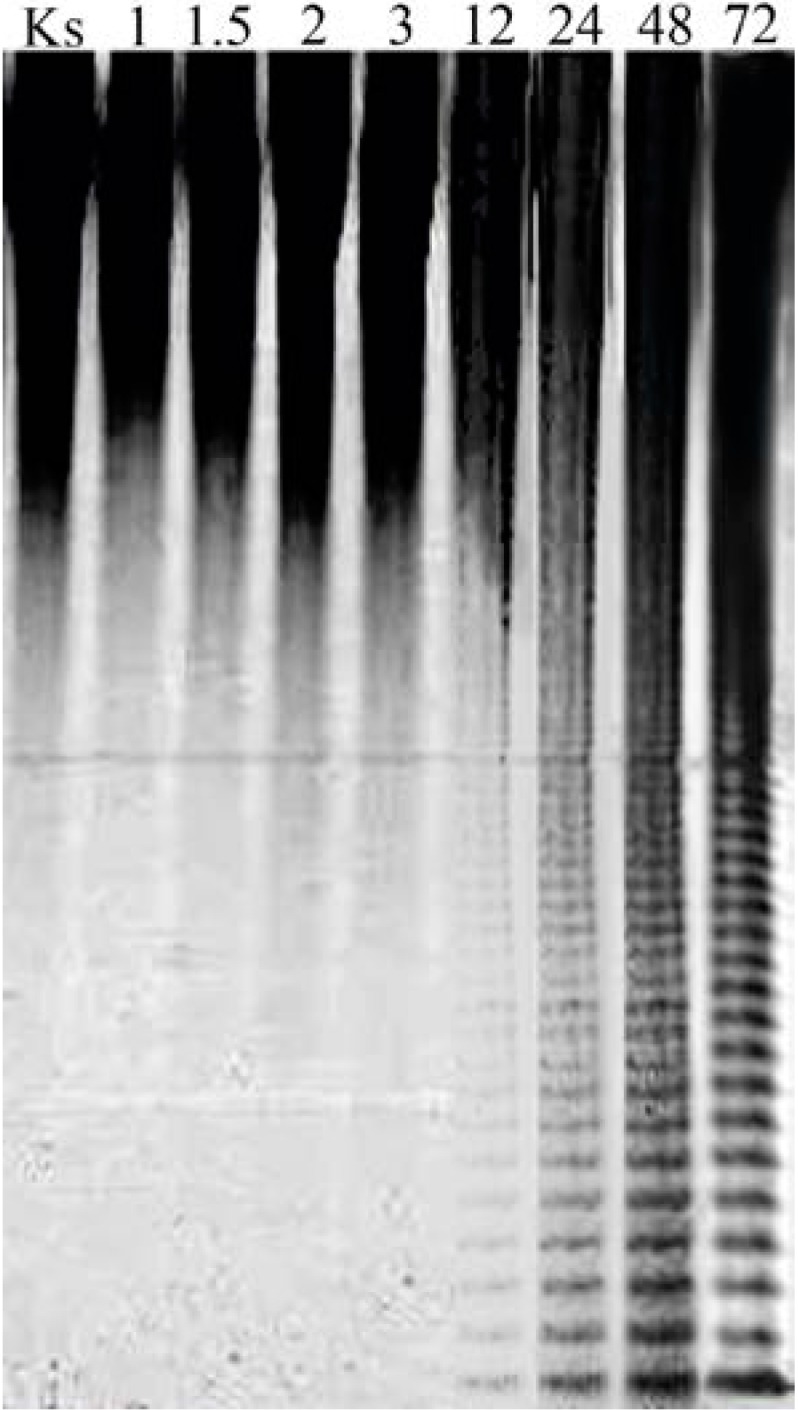
Electropherogram of the fucoidan fragments produced by FFA at different incubation times. The numbers above the lines refer to the time of incubation in hours. Ks—unhydrolyzed fucoidan.

We studied the kinetics of *F. evanescens* fucoidan hydrolysis by FFA. The sulfated oligosaccharides were identified on an electropherogram after 12 h of incubation, and the maximum yield of fucoidan enzymatic hydrolysis products was observed after 48 h (Figure 4).

FFA had greater temperature stability than the fucoidanase from *Haliotis* sp. [32]; however, it was less stable than the fucoidanase isolated from the marine bacterium, *Vibrio* sp. N-5 [38].

**Figure 5:**
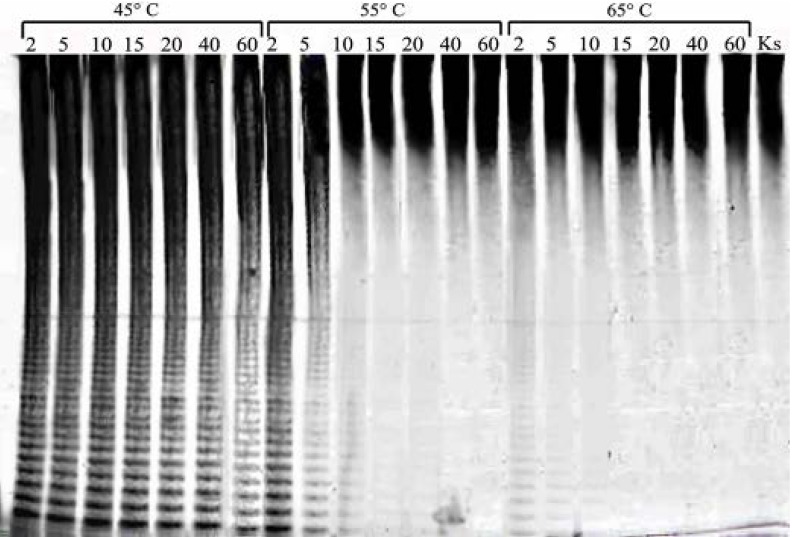
Electropherogram of the fucoidan fragments produced by FFA at different incubation temperature. The number above the top of the gel refers to the time of FFA preincubation in minutes. The preincubation temperature is indicated over the brackets. Ks—unhydrolyzed fucoidan.

The enzymatic activity of FFA was considerably reduced after prolonged (about 60 min) incubation of the enzyme solution at 45 °C. The enzyme was completely inactivated after 40 min of incubation at 55° C (Figure 5). The most important properties of enzymes are the specificity and mode of action, which are the basis for their further use in structural studies and biotechnological processes. Most of the previously studied fucoidanases were endo-type enzymes that could cleave the glycosidic bonds within a molecule of fucoidan and produce oligosaccharides with different degrees of polymerization [34,40]. Unsulfated oligosaccharides were not observed in the reaction products. Only three exo-type fucoidanases have been isolated from the marine bacterium, *Vibrio* sp. N-5. The main products of these fucoidanases were sulfated fucose and sulfated fucobioses [38].

It is evident from the electropherogram that a portion of the substrate is resistant to hydrolysis. Apparently, FFA has specificity not only for the type of cleavable bond, but also for more fine elements of fucoidan structure, such as the position of the sulfate groups. 

Fucoidanase isolated from *F. algae* catalyzed the hydrolysis of fucoidans from *F. evanescens* and *F. vesiculosus*; however, it did not hydrolyze fucoidan from *S. cichorioides* or the sulfated galactan—carrageenan (Table 2). Desulfated fucoidan from *F. evanescens* was hydrolysed very weakly in contrast to deacetylated fucoidan, which was hydrolysed more actively compared to the natural fucoidans from *F. evanescens*.

**Table 2:** Specificity of FFA action on different substrates.

Substrate	Yield of hydrolysis products, %
Fucoidan from *F. evanescens*	7
F. dAc*	9.4
F. dS*	0.8
Fucoidan from *F. vesiculosus*	5.6
Carrageenan	0
Fucoidan from *S. cichorioides*	0

F. dAc*, deacetylated derivative of *F. evanescens* fucoidan; F. dS*, desulfated derivative of *F. evanescens* fucoidan.

Three samples of fucoidans with different structural characteristics were isolated from the brown algae, *F. evanescens*, *F. vesiculosus* and *S. cichorioides*, and were used as substrates to study the specificity of the fucoidanase. It was shown that the fucoidanase did not catalyze the hydrolysis of fucoidan from *S. cichorioides*, which contains only α-1→3 glycosidic bonds. Fucoidans from *F. evanescens* and *F. vesiculosus* containing alternating α-1→4 and α-1→3 glycoside bonds were hydrolysed by the enzyme. From these data, we can conclude that the FFA is specific for the α-1→4 glycosidic bonds. We also investigated whether FFA could catalyze the cleavage of linkages in chemically modified fucoidan from *F. evanescens* (Table 2). It is clear that sulfate groups have a strong influence on enzymatic activity, because the yield of low-molecular weight products was very low in the case of the desulfated derivative. The presence of sulfate groups was essential for the action of the FFA, most likely because of binding between the enzyme and substrate. In contrast, deacetylated fucoidans have an increased yield of low-molecular weight products. Removing the acetate groups seemed to make the glycosidic linkages more available for the action of FFA.

For a detailed investigation of the specificity of fucoidanase, FFA products from fucoidan from *F. evanescens* were obtained. NMR spectra of highly purified fucoidan, deacetylated and desulfated derivatives corresponded closely to previously published NMR spectra [7]. Thus, our fraction of fucoidan has a linear backbone of alternating three- and four-linked α-l-fucopyranose 2-*O*-sulphate residues: →3)Fuc2SO_3_-(1→4)Fuc2SO_3_-(1→. An additional sulfate occupies position 4 in a portion of the three-linked fucose residues, whereas a portion of the remaining hydroxyl groups is randomly acetylated. The monosaccharide composition of the purified fucoidan was shown to be fucose, galactose and xylose, with a molar ratio of 1, 0.03 and 0.01, respectively. The molar ratio of Fuc:SO_3_Na was 1:1.2.

**Figure 6:**
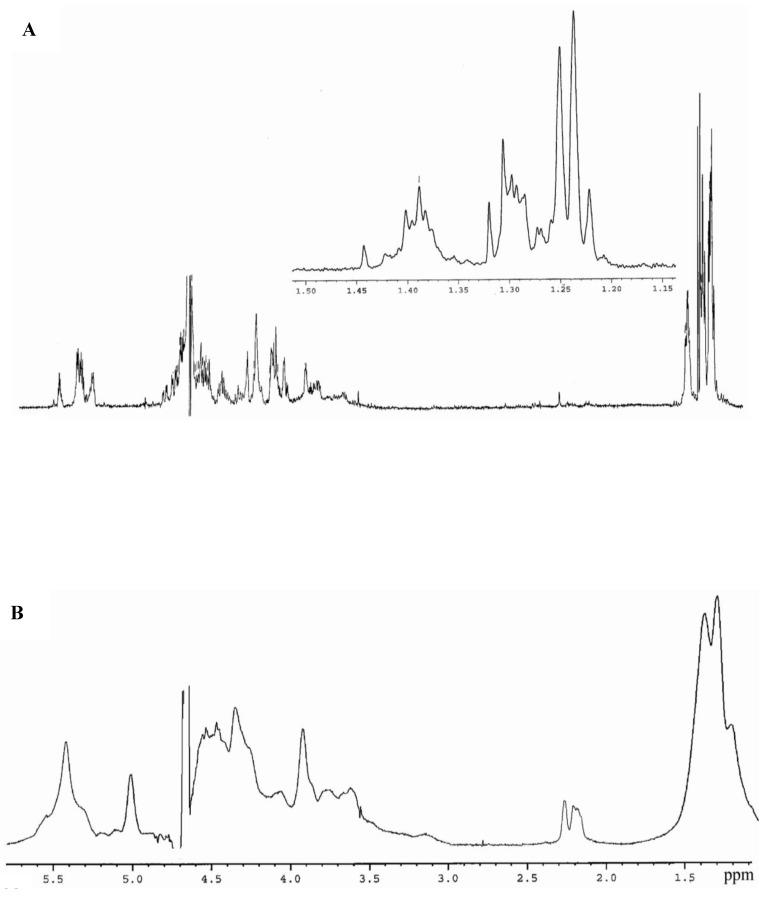
(**A**) ^1^H NMR spectrum of FFA enzymatic hydrolysis products of fucoidan (the low molecular weight products (LMP)) from *F. evanescens*; (**B**) ^1^H NMR spectrum of native fucoidan from *F. evanescens*.

The high molecular weight products of enzymatic hydrolysis (HMP) were separated from the low molecular weight products (LMP) by precipitation with 75% aqueous ethanol. The yield of LMP was 49%. The ^1^H NMR spectrum of the LMP was different from the native fucoidan (Figure 6), particularly in the regions of the α-anomeric protons (5.6–5.3 ppm) and the H6 protons of the methyl groups (1.4–1.2 ppm). We have estimated the integral intensity of signals at an area of 1.24–1.32 ppm and 1.38–1.41 ppm, and the ratio of 1→3- and 1→4-links in the LMP was compared. The same method was used earlier by R. Daniel [29]. Using this calculation, the amount of 1→3:1→4 in the fucoidan prior to the action of the enzyme was approximately equal. After the enzymatic reaction, the corresponding ratio was approximately 3:1. The H4 signal (4.9 ppm) corresponding to fragment, →3)Fuc2,4diSO_3_^−^(1→, was absent in the LMP ^1^H spectrum. It appears that the fucoidanase from *F. algae* cleaves the (1→4)-linkages within →3)Fuc2,4diSO_3_^−^-(1→4)Fuc2SO_3_^−^-(1→ of the fucoidan backbone.

Therefore, the marine bacterium, *F. algae*, synthesized an α-l-fucanase with an endo-type of action. The enzyme is specific for hydrolyzing the 1→4-bonds that neighbor Fuc2,4diSO_3_^−^ residues.

**Figure 7:**
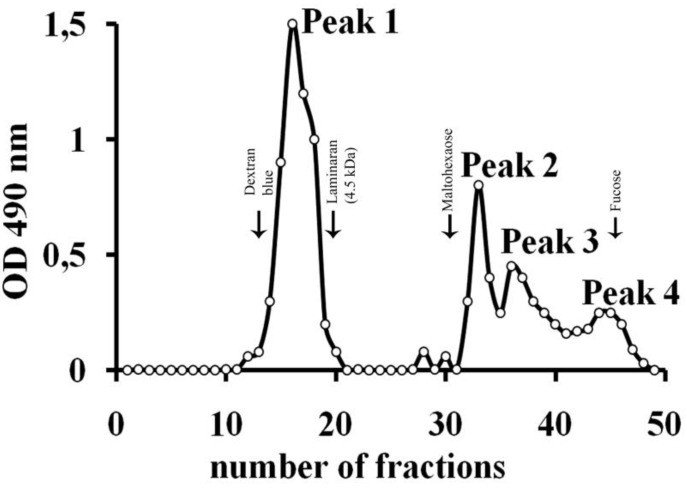
Gel filtration of the LMP fraction on a Bio-Gel P-6 column.

Then, the LMP fraction was fractionated by gel-filtration on Bio-gel P-6 (Figure 7) and Bio-gel P-2 (data not shown). Only for fraction “Peak 2” after rechromatography on Bio-gel P-2 (Figure 7), both ^1^H and ^13^C NMR spectra were resolved enough for detailed investigation by NMR spectroscopy.

The assignment of the major peaks was achieved by analysis of 1D ^1^H, ^13^C NMR spectra and 2D spectroscopy. COSY and HSQC NMR spectra of the “Peak 2” featured chemical shifts assigned to four different α-l-fucopyranosyl residues, referred to as A, B, C and D (Table 3). Two-dimensional COSY, TOCSY, HSQC and HMBC techniques were used for assignment of the signals of all four residues, as summarized in Table 3. The position of sulfate groups in “Peak 2” was deduced from the downfield shifts of H2 on Δδ = 0.7 ppm [27]. It means that all fucose residues were sulfated at C2. H3 signals of fucose residue C with a chemical shift of 4.7 ppm could be assigned to fucose sulfated at C3 [24]. Cross-peaks in the HMBC spectrum between anomeric protons (5.32 ppm) of residue A and C3 (73.9 ppm) of residue B, H1 (5.34 ppm) of residue C and C3 (75.2 ppm) of residue D indicate the presence of α-1→3-linkages. The ratio of integral intensity of the signals of anomeric protons of residue A and residue C was 1:0.5. From these data, we can conclude that we obtained a mixture of disaccharides with a ratio of 2:1 following structures α-l-Fuc*p*2SO_3_^−^(1→3)-α-l-Fuc*p*2SO_3_^−^ and α-l-Fuc*p*2,3SO_3_^−^(1→3)-α-l-Fuc*p*2SO_3_^−^. It should be noted that the latter fragment was detected previously in the native fucoidan from *F. evanescens*, probably due to its low content, although the presence of 2,3-disulphated fucose residues was supposed in several closely related polysaccharides [9].

**Table 3:** Chemical shifts (ppm) for the “Peak 2” oligosaccharides.

**Residue**	**H1**	**H2**	**H3**	**H4**	**H5**	**H6**
A	5.32	4.45	4.08	3.88	4.22	1.23
B	5.48	4.50	4.02	4.06	4.47	1.22
C	5.34	4.56	4.70	4.21	4.53	1.24
D	5.50	4.51	4.03	4.08	4.48	1.23
**Residue**	**C1**	**C2**	**C3**	**C4**	**C5**	**C6**
A	95.3	76.4	69.9	73.3	67.1	16.0
B	91.7	74.6	73.9	68.6	67.9	16.1
C	96.4	73.6	76.2	71.7	67.8	16.3
D	91.7	74.6	75.2	70.5	67.8	16.2

Previously, it was shown that fucoidan from *F. evanescens* induced maturation of dendritic cells and stimulated humoral and cellular immune response, as well as functional activity of neutrophils and macrophages [41,42].

The process of lymphocyte activation leads to expression of activation antigens. CD69 is one of the earliest cell surface antigens expressed by T-cells following activation. Once expressed, CD69 acts as a costimulatory molecule for T cell activation and proliferation. In addition to mature T-cells, CD69 is inducibly expressed by immature thymocytes, B-cells, natural killer (NK) cells, monocytes, neutrophils and eosinophils and is constitutively expressed by mature thymocytes and platelets. 

CD25, a component of the IL-2 receptor, is important in T-cell proliferation, activation induced cell death, as well as the actions of both regulatory and effector T-cells and have a better antigen-presenting capacity.

We investigated the expression level of activation antigens, CD25 and CD69, involved in adaptive and innate immunity induced by the native fucoidan and the products of its enzymatic transformation.

It was shown that the native fucoidan, as well as HMP and “Peak 2” induced a significant increase of the number of CD3+-lymphocytes (T-lymphocytes) and CD16+-cells (NK + NKT-lymphocytes) that express the activation molecules, CD69 and CD25. All the studied polysaccharides activated CD16+-cells to a greater extent than CD3+-cells (Table 4).

Products of the enzymatic transformation, as well as native fucoidan retained the ability to activate cells of the immune system despite the different structure.

**Table 4:** Influenceof fucoidanon the expression of early activation antigens on peripheral blood lymphocytes.

Lymphocyte activation marker	Control M ± σ	Fucoidan from *F. evanescens* M ± σ	HMP M ± σ	“Peak 2” M ± σ
CD16^+^ CD69^+^ (percentage of CD16^+^-lymphocytes)	33.4 ± 11.9	88.1 ± 6.5 *p* = 0.000	93.2 ± 4.4 *p* = 0.000	90.8 ± 5.1 *p* = 0.000
CD16^+^ CD25^+^ (percentage of CD16+-lymphocytes)	2.8 ± 2.0	17.4 ± 5.89 *p* = 0.000	21.9 ± 4.9 *p* = 0.000	19.3 ± 5.9 *p* = 0.000
CD3^+^ CD69^+^ (percentage of CD3^+^-lymphocytes)	14.9 ± 5.2	25.3 ± 12.9 *p* = 0.008	30.1 ± 13.0 *p* = 0.000	37.1 ± 15.3 *p* = 0.002
CD3^+^ CD25^+^ (percentage of CD3^+^-lymphocytes)	11.8 ± 3.1	16.6 ± 3.9 *p* = 0.000	19.2 ± 3.2 *p* = 0.000	19.1 ± 3.0 *p* = 0.000

Note: *p* < 0.05 compared with the control group; HMP, high molecular weight products of enzymatic hydrolysis.

## 3. Experimental Section

### 3.1. Reagents

Crude fucoidans from *Saccharina cichorioides* (*Laminaria* *cichorioides*) and *Fucus evanescens* were obtained as described by Zvyagintseva *et al.* [43]. The fucoidans were then purified by ion-exchange chromatography, as described previously [44]. Desulfated fucoidans from *F. evanescens* were prepared as described [7], and deacetylated fucoidans from *F. evanescens* were prepared as described [11]. Laminarin from *S. cichorioides* and pustulan from *Umbilicaria russica* were obtained as described [42,45]. Crude fucoidan from *F. vesiculosus*, alginic acid, κ/λ carrageenan, amylopectin and CM-cellulose were purchased from Sigma (St. Louis, MO, USA).

### 3.2. General Methods

The total amount of carbohydrate was determined by the phenol-sulfuric acid method with l-fucose as the standard [46]. The amount of sulfate residues was determined using the BaCl_2_-gelatine method, with Na_2_SO_4 _as the standard. The monosaccharide composition of the fucoidans was determined after acid hydrolysis of the samples with 2 M trifluoroacetic acid (100 °C, 7 h) by HPLC (column: Shim-pack ISA-07/S2504; 0.4 × 25 cm; 70 °C, Shimadzu, Tokyo, Japan) with detection using the bicinchoninate method. The monosaccharides, rhamnose (Rha), mannose (Man), fucose (Fuc), galactose (Gal), xylose (Xyl) and glucose (Glc), were used as the standards.

The protein concentration was determined by the method of Bradford [47], with bovine serum albumin as the standard.

### 3.3. Enzyme Activity Assay

The reaction mixture for glycanase activity determination contained 200 μL of 0.1% substrate solution comprised of either alginic acid, amylopectin, CM-cellulose, laminarin or pustulan and 50 μL of the enzyme in phosphate buffer, pH 7.2. The reaction was performed at 37 °C. The activity was determined by measuring the amount of reducing sugar released from the polysaccharides using the Nelson method, with glucose as the standard [48]. One unit of glycanase activity (U) was defined as the quantity of enzyme catalyzing the formation of 1 μmol of glucose in 1 min under standard conditions.

The activity of the glycosidases was determined using *p*-nitrophenyl derivatives of the corresponding sugars as substrates. One unit of enzyme activity (U) was defined as the quantity of enzyme catalyzing the formation of 1 μmol of *p*-nitrophenol in 1 min under standard conditions. 

The activity of the fucoidanase was monitored by carbohydrate-polyacrylamide gel electrophoresis (C-PAGE). The fucoidanase activity was detected by the occurrence of charged oligosaccharide bands in the gel. The reaction mixture containing 50 μL of enzyme solution in 0.015 M phosphate buffer, pH 7.2, and 100 μg of fucoidans from *F. evanescens*, *F. vesiculosus* or *S. cichorioides* was incubated at 37 °C for 24 h. The reaction was stopped by freezing. The hydrolysis products were mixed with 10 μL loading buffer containing a 10% solution of glycerol in water and 0.02% phenol red. The samples were electrophoresed through a 5% (w/v) stacking gel with 50 mM Tris-HCl buffer pH 6.8 and 27% (w/v) resolving polyacrylamide gel with 150 mM Tris-HCl buffer pH 8.8. The gel was 1 mm thick. Gel staining was performed with a solution containing 0.01% *O*-toluidine blue in EtOH, AcOH and H_2_O with a volume ratio of 2:1:1.

### 3.4. Bacterial Culture Conditions

*F. algae* strain KMM 3553^T^ was isolated from the brown algae, *F. evanescens* [37], and was grown for 24 h at 28 °C on medium containing 0.5% (w/v) Bacto peptone (Difco), 0.1% (w/v) Bacto yeast extract (Difco), 0.1% (w/v) glucose, 0.02% (w/v) KH_2_PO_4_, 0.005% (w/v) MgSO_4_·7H_2_O, 50% (v/v) natural seawater and 50% (v/v) distilled water, pH 7.8. The bacterial biomass production during growth in liquid medium was evaluated by measuring the culture precipitate after centrifugation at 4500× *g* for 30 min.

Fucoidanase activity during growth of the bacteria was monitored by C-PAGE under the following conditions: 1 g of the bacterial biomass was poured into 3 mL of 0.01 M phosphate buffer pH 7.2, and the resuspended bacteria were sonicated for 2 min. The extract was then centrifuged at 10,000× *g* for 30 min, and a 100 µL aliquot of the extract was removed for C-PAGE estimation of the fucoidanase activity, as described above.

### 3.5. NMR Spectroscopy

The ^1^H NMR spectra were recorded at 700 MHz at 35 °C using an Avance 700 Bruker spectrometer (Bruker BioSpin GmbH, Rheinstetten, Germany). The samples were dissolved in D_2_O, and acetone was used as the standard. The data were acquired and performed using Topspin 2.1 version (Bruker BioSpin GmbH, Rheinstetten, Germany). The parameters used for 2D experiments were as follows: COSY (2048 × 256 data matrix; zero-filled to 2048 data points in *t*_1_; 16 scans per *t*_1_ value; spectral width of 4550 Hz; recycle delay of 1 s); TOCSY (2048 × 256 data matrix; zero-filled to 2048 data points in *t*_1_; 64 scans per *t*_1_ value; the duration of the MLEV17 spin-lock was 60 ms); HSQC (2048 × 512 data matrix; zero-filled to 512 data points in *t*_1_; 64 scans per *t*_1_ value; spectral width in *t*_1_, 2400 Hz, and in *t*_2_, 29,900 Hz; recycle delay of 1 s; shifted sine-squared filtering in *t*_1_ and *t*_2_); HMBC (4096 × 256 data matrix; 64 scans per *t*_1_ value; spectral width in *t*_2_, 6300 Hz, and *t*_2_, 31,690 Hz; recycle delay of 1 s; optimization of the experiment for coupling constant, 8 Hz).

### 3.6. Purification of Fucoidanase

All purification steps were performed at 4 °C. After fermentation, the cells were collected by centrifugation for 30 min at 4500× *g*. The working buffer, 0.01 M phosphate buffer, pH 7.2, was added to 15 g of the bacterial biomass. This mixture was disrupted by sonication at 20 kHz three times for 20 s each. The suspension was centrifuged at 9000× *g* for 30 min to remove the cellular debris. The supernatant (40 mL) was applied to a DEAE-MacroPrep column (3 × 10 cm, Bio-Rad, Hercules, CA, USA) equilibrated with the same buffer as above. The proteins were eluted with a linear gradient of 0 to 0.5 M NaCl in a total working buffer volume of 300 mL and a flow rate of 0.7 mL/min. The fractions with fucoidanase activity were concentrated by ultrafiltration through a 10-kDa molecular weight cut-off membrane to a final volume of 0.5 mL. These concentrated fractions were then applied to a Sephacryl S-200 column (1 × 70 cm, GE Healthcare, Uppsala, Sweden). The fractions with fucoidanase activity were pooled and used for investigation.

### 3.7. Determination of the Optimum pH for Fucoidanase

The reaction mixture containing 100 µL of fucoidanase solution, 200 µg *F. evanescens* fucoidan and 50 µL of buffers with various pH values (0.2 M citrate-phosphate buffers with pH values ranging from 3.0 to 8.5 or borate buffers, pH 9.0) were incubated for 24 h at 37 °C. The activity was monitored by C-PAGE, as described above.

### 3.8. Determination of Fucoidanase Stability at Different Temperatures

The fucoidanase solution in 0.015 M phosphate buffer pH 7.2 was incubated at 20, 37, 45, 50, 55 and 65 °C, and aliquots were collected at 2, 5, 10, 15, 20, 40 and 60 min. A sample of the fucoidanase was cooled, and the substrate was then added. The activity was monitored as described above.

### 3.9. Determination of the Yield of the Low Molecular Weight Products

The reaction mixture containing 500 µL of fucoidanase solution and 3 mL (10 mg/mL) of substrate (fucoidan, fucoidan derivatives and carrageenan) solution in working buffer was incubated for 24 h at 37 °C. The high molecular weight products (HMP) were precipitated with ethanol, which was added to the solution at a ratio of 1:3 (v/v). This mixture was then centrifuged at 9000× *g* for 20 min. The supernatant containing the low molecular weight products (LMP) was evaporated, and the resulting products were dissolved in 1 mL of water. The yield of the low molecular weight products was calculated as the ratio of the sugars content in the low molecular weight products to the total content of sugars in the reaction mixture expressed as a percentage. The content of the sugars was analyzed by the phenol-sulfuric method [44], and fucose was used as the standard. 

### 3.10. Preparation of Fucoidan Oligosaccharides

Fucoidan from *F. evanescens* (300 mg) was dissolved in 16 mL of the partially purified enzyme fraction (after ion exchange column) in 0.01 M sodium-phosphate buffer, pH 7.2, with the addition of 15 mM NaCl and incubated at 37 °C for 72 h. The reaction mixture was then deproteinized by heating at 80 °C for 10 min, and the precipitate was removed by centrifugation. The high molecular weight reaction products (HMP) were precipitated with ethanol that was added at a ratio of 1:3 (v/v), and the precipitate was isolated by centrifugation at 9000× *g* for 20 min. The supernatant containing the low molecular weight reaction products (LMP) were concentrated under a vacuum. The LMP were then applied onto a Biogel P-6 column (1 × 120 cm, Bio-Rad, Hercules, CA, USA) equilibrated with water. The fractions containing the carbohydrates were pooled and concentrated by vacuum evaporation. Fractions “Peak 2”, “Peak 3” and “Peak 4” were rechromatographed on a Biogel P-2 column (1 × 120 cm) equilibrated with water. The fractions containing carbohydrates were pooled and concentrated by vacuum evaporation and then freeze-dried. The carbohydrates in the fractions were detected with the phenol-sulfuric method [46].

### 3.11. Influence of Bivalent Metals

Fucoidan (200 µg) was added to a mixture comprised of 100 µL of the fucoidanase and one of the following solutions: 0.25 M BaCl_2_, CaCl_2_, MgCl_2_, ZnCl_2_ or CuSO_4_ (20 µL). The mixture was then incubated for 24 h at 37 °C, and the activity was determined by C-PAGE analysis.

### 3.12. Kinetics of the Enzymatic Reaction

The reaction mixture containing 600 µL of the fucoidanase solution in the working buffer and 1.2 mg of fucoidan was incubated at 37 °C for 1, 1.5, 2, 3, 12, 24, 48 and 72 h. Aliquots (50 µL) of the reaction mixture were removed after incubation, and the enzyme activity was monitored by C-PAGE.

### 3.13. Flow Cytometric Measurement of CD69 and CD25

The material for the study was the peripheral blood samples with heparin obtained from nine healthy donors. Samples were dissolved in saline (0.9% NaCl) and introduced into the blood samples in the final concentration, corresponding to 100 µg/mL of carbohydrates.

The level of expression of activation markers, CD69 and CD25, on the surface of CD16+- and CD3+-lymphocytes was determined after 24 h of incubation with fucoidan by two-color cytometric analysis using the program “Cell Quest” by the flow cytometer, “FACSCalibur” (“Becton Dickinson”, USA), with the monoclonal antibodies to the CD16-FITC, CD3-FITC, CD69-PE and CD25-PE molecules (“Beckman Coulter”) and the corresponding isotypic controls.

Statistical analysis was performed using the software package, Statistica 7.0 (StatSoft, Tulsa, OK, USA), by the Student’s *t*-test. 

## 4. Conclusions

Marine bacterium *Formosa algae* strain KMM 3553 synthesized an α-l-fucanase with an endo-type action that is specific for 1→4-bonds in a molecule of the fucoidans. Fucanase from *F. algae* can produce the sulfated fuco-oligosaccharides, which possess immunomodulating activity. In our opinion, the main problem for fucoidanases investigation is the absence of quantitative methods for fucoidanase assay. Nevertheless, investigation of fucoidanases is of special interest. Enzymatic depolymerization provides an indispensable tool, not only for production of fuco-oligosaccharides, which might display a wide spectrum of biological functions, but also for structural studies of fucoidans.
